# Media use trajectories and risk of metabolic syndrome in European children and adolescents: the IDEFICS/I.Family cohort

**DOI:** 10.1186/s12966-021-01186-9

**Published:** 2021-10-18

**Authors:** Elida Sina, Christoph Buck, Toomas Veidebaum, Alfonso Siani, Lucia Reisch, Hermann Pohlabeln, Valeria Pala, Luis A. Moreno, Dénes Molnar, Lauren Lissner, Yiannis Kourides, Stefaan De Henauw, Gabriele Eiben, Wolfgang Ahrens, Antje Hebestreit, the IDEFICS, I.Family consortia

**Affiliations:** 1 Leibniz Institute for Prevention Research and Epidemiology-BIPS, Achterstraße 30, 28359 Bremen, Germany; 2 Department of Chronic Diseases, National Institute for Health Development, Tallinn, Estonia; 3 Institute of Food Sciences, National Research Council, Avellino, Italy; 4 Department of Management, Society and Communication, Copenhagen Business School, Copenhagen, Denmark; 5 Department of Preventive and Predictive Medicine, Fondazione IRCCS, Istituto Nazionale Dei Tumori, Milan, Italy; 6 GENUD (Growth, Exercise, Nutrition and Development) Research Group, Instituto Agroalimentario de Aragón (IA2), Instituto de Investigación Sanitaria Aragón (IIS Aragón), Centro de Investigación Biomédica en Red Fisiopatología de La Obesidad Y Nutrición (CIBERObn), University of Zaragoza, Zaragoza, Spain; 7 Department of Pediatrics, Medical School, University of Pécs, Pécs, Hungary; 8 School of Public Health and Community Medicine, University of Gothenburg, Gothenburg, Sweden; 9 Research and Education Institute of Child Health, Strovolos, Cyprus; 10 Department of Public Health and Primary Care, Ghent University, Ghent, Belgium; 11 Department of Public Health, School of Health Sciences, University of Skövde, Skövde, Sweden; 12 Institute of Statistics, Faculty of Mathematics and Computer Science, University of Bremen, Bremen, Germany

**Keywords:** Longitudinal study, Digital media, Screen-time, Metabolic disorders, Sedentary behavior, Physical activity, Diet quality, Children, Adolescents

## Abstract

**Background:**

Media use may influence metabolic syndrome (MetS) in children. Yet, longitudinal studies are scarce. This study aims to evaluate the longitudinal association of childhood digital media (DM) use trajectories with MetS and its components.

**Methods:**

Children from Belgium, Cyprus, Estonia, Germany, Hungary, Italy, Spain and Sweden participating in the IDEFICS/I.Family cohort were examined at baseline (W1: 2007/2008) and then followed-up at two examination waves (W2: 2009/2010 and W3: 2013/2014). DM use (hours/day) was calculated as sum of television viewing, computer/game console and internet use. MetS z-score was calculated as sum of age- and sex-specific z-scores of four components: waist circumference, blood pressure, dyslipidemia (mean of triglycerides and HDL-cholesterol^−1^) and homeostasis model assessment for insulin resistance (HOMA-IR). Unfavorable monitoring levels of MetS and its components were identified (cut-off: ≥ 90^th^ percentile of each score). Children aged 2–16 years with ≥ 2 observations (W1/W2; W1/W3; W2/W3; W1/W2/W3) were eligible for the analysis. A two-step procedure was conducted: first, individual age-dependent DM trajectories were calculated using linear mixed regressions based on random intercept (hours/day) and linear slopes (hours/day/year) and used as exposure measures in association with MetS at a second step. Trajectories were further dichotomized if children increased their DM duration over time above or below the mean.

**Results:**

10,359 children and adolescents (20,075 total observations, 50.3% females, mean age = 7.9, SD = 2.7) were included. DM exposure increased as children grew older (from 2.2 h/day at 2 years to 4.2 h/day at 16 years). Estonian children showed the steepest DM increase; Spanish children the lowest. The prevalence of MetS at last follow-up was 5.5%. Increasing media use trajectories were positively associated with z-scores of MetS (slope: β = 0.54, 95%CI = 0.20–0.88; intercept: β = 0.07, 95%CI = 0.02–0.13), and its components after adjustment for puberty, diet and other confounders. Children with increasing DM trajectories above mean had a 30% higher risk of developing MetS (slope: OR = 1.30, 95%CI = 1.04–1.62). Boys developed steeper DM use trajectories and higher risk for MetS compared to girls.

**Conclusions:**

Digital media use appears to be a risk factor for the development of MetS in children and adolescents. These results are of utmost importance for pediatricians and the development of health policies to prevent cardio-metabolic disorders later in life.

**Trial registration:**

ISRCTN, ISRCTN62310987. Registered 23 February 2018- retrospectively registered.

**Supplementary Information:**

The online version contains supplementary material available at 10.1186/s12966-021-01186-9.

## Background

Non-communicable diseases have reached alarming proportions worldwide [1]. Cardiovascular diseases in adults are associated with cardio-metabolic factors including hypertension, dyslipidaemia, abdominal obesity and abnormal glucose regulation- the combination of which is known as metabolic syndrome (MetS) [2]. These associations are observed in children as well [3]. Cohort studies have shown that childhood MetS is associated with a 2.4 fold risk of MetS in adulthood [4].

Unhealthy eating, low levels of physical activity and sedentary behaviours (SB), the latter characterized by activities that require low energy expenditure performed in reclining or lying position such as sitting in front of screens, substantially contribute to the development of MetS [5]. World Health Organisation (WHO) has recognized the adverse role of prolonged exposure to digital media (DM) in childhood obesity and recommends that children and adolescents should limit recreational screen-time [6]. Remarkably, current evidence suggests that average screen-time (excluding school-related work) stands at 5 h/day in children and 8 h/day in adolescents [7]. This underlines the displacement of physical activity in favour of screen-based activities which may associate with adverse health outcomes.

Cross-sectional studies have reported a positive relationship between screen-media exposure and metabolic disorders in obese children [8–11]. Prolonged television viewing (TV) has been associated with obesity throughout the life course [12], but represents only one component of screen-time. With increasing age, TV is displaced by other digital media (e.g. computer use) which provide access to internet platforms [13]. Thus, it is important to evaluate the combined impact of these media types on the development of MetS, particularly in youth.

Evidence on the longitudinal associations between DM use and MetS in children and adolescents is currently lacking. Hence, based on the definition of childhood MetS developed by Ahrens et al. (2014) [14], we aim to investigate the longitudinal association of DM use during childhood with incident MetS and its components, including abdominal obesity, dyslipidaemia, insulin resistance (IR) and high blood pressure (BP) at two to six years after baseline examination in European children and adolescents. We use a trajectory analysis approach while taking into account sex and country discrepancies. Moreover, in a sample with available accelerometer data, we investigate the confounding role of objectively-measured sedentary time (SED) and moderate to vigorous physical activity (MVPA) in the association between DM use and MetS.

## Methods

### Design

The IDEFICS/I.Family cohort includes children and adolescents from 8 European countries: Belgium, Cyprus, Estonia, Germany, Hungary, Italy, Spain and Sweden. During 2007–2008, 16,229 children aged 2–9 years, meeting the basic inclusion criteria (complete information on age, sex, weight and height; attending kindergartens or grade 1 and 2 of primary schools and residing in the respective regions) participated in baseline (W1) [15]. In the second wave (W2: 2009–2010), 13,596 children were re-examined (68% of W1 (11,041); 2555 children were newly recruited from new families)). The I.Family study (2013–2014) represents the third wave (W3), where 9,617 children and (meanwhile) adolescents aged 2–17 years were re-examined: 73.8% of them already participated at W2 (7105) while 2512 were new children (siblings from the same families) [15, 16]. Informed consent was obtained from adolescents (≥ 12 years), and the assent was given from younger children, in addition to parental informed consent, at all study waves. Ethical approval was obtained from local ethic committees of each study center.

### Participants

The cohort includes 21,272 children and adolescents who participated at baseline and/or at one or two follow-up examinations (W1/W2; W1/W3; W2/W3; W1/W2/W3), accounting for 39,433 observations in total. Observations excluded were those with implausible age at follow up (*N* = 6), implausibly high DM use (> 50 h/week, *N* = 137), missing information on DM (*N* = 3,240) and all metabolic risk-factors (*N* = 1,031); aged > 16 years or being non-fasting during blood sampling (*N* = 1,745); suffering from chronic diseases (e.g. MetS, Type 2 Diabetes) at baseline or taking related medications (W1: *N* = 131; W2: *N* = 204; W3: *N* = 15). The analysis group was restricted to children participating in ≥ 2 examination waves, leading to a final analysis population of 10,359 children (24,075 observations in total; 41.8% contributed three observations (i.e. 10,071 observations of 3357 children). The observation period ranged between 2 to 6 years (median = 5 years) as children could enter the cohort at W1 in 2007/08 or at W2 in 2009/10 (median age = 6.3 years, (IQR = 4.5–7.6 years)) and were then followed up until W3 in 2013/14. The median age at last follow-up was 10 years (IQR = 8.6–12.2 years).

### Media use

DM use was proxy-reported by parents of children aged < 12 years and self-reported by adolescents, using respectively a parental and a teen version of the core questionnaire, pre-tested for reliability and acceptability [17]. Information on TV and computer/game console use was separately reported for weekdays and weekend days in all waves as: “Not at all, < 30 min/day, < 1 h/day, 1–2 h/day, 2–3 h/day, > 3 h/day”. At W3, duration of internet use was additionally provided as a proxy for the exposure to online games and online advertisements for unhealthy foods. Total DM use was calculated as sum of the weighted average of durations reported for weekdays and weekend days (minutes/week) at all waves for all screen-time behaviors (TV, PC and internet use for W3), and quantified as hours/day. Hereinafter, the terms DM use and screen-time will be interchangeably used.

### Clinical and laboratory evaluations

Blood pressure (BP) was measured in children after resting for 5 min in a sitting position using an automated oscillometric device (Welch Allyn 4200B-E2, Welch Allyn Inc., New York, NY, USA) [18]. The average of two measurements [19] was calculated for the analysis. Waist circumference (WC) was measured according to the international standards of kinanthropometry [20]. Fasting blood samples were collected and levels of glucose, insulin, high-density lipoprotein cholesterol (HDL-c) and triglycerides were measured [14]. The Homeostasis Model Assessment for Insulin Resistance (HOMA-IR) was calculated as (fasting insulin*fasting glucose)/405 [21]. Age and sex-specific z-scores were derived for children and adolescents aged 2–16 years for WC [22], HDL-c, triglycerides [23], diastolic and systolic BP (and height-specific) [19] and HOMA-IR [21].

### Metabolic syndrome

A continuous score for cardio-metabolic risk has been proposed by Ahrens et al. (2014) [14], to combine the four components of MetS. The additive MetS score was calculated as sum of z-scores of HOMA-IR, WC, BP (mean of age-, sex- and height-specific z-scores of diastolic and systolic BP), and dyslipidemia (mean of z-scores of triglycerides and HDL-c, the latter multiplied with -1 due to the inverse relationship with the metabolic risk).

A monitoring level for MetS [14] was defined if at least three of the four MetS components exceeded the 90^th^ percentile of the respective age- and sex-specific distributions. Unfavorable levels of the four components were identified (monitoring level: ≥ 90^th^ percentile): abdominal obesity measured via WC, IR measured via HOMA-IR or fasting insulin; hypertension via diastolic or systolic BP and dyslipidemia via triglycerides or HDL-c (≤ 10^th^ percentile). Subsequently, children being at the monitoring level for MetS and its components were considered as requiring closer monitoring by the clinician. For clarity, the terms MetS, abdominal obesity, elevated BP, dyslipidemia and IR will be respectively used to refer to the monitoring level for each metabolic outcome.

### Potential confounders

Using a food frequency questionnaire—previously tested for relative validity and reproducibility [24, 25], participants reported the consumption frequency of unhealthy snacks (times/week) during the past four weeks (self-reported by adolescents or proxy reported by parents of younger children), including sugar-sweetened drinks, chocolate/nut-based spread, crisps, corn crisps and popcorn, chocolate/candy bars, candies, loose candies and marshmallows. The median of daily consumption frequency was calculated and categorized as high vs. low snack intake. In addition, a healthy diet adherence score (HDAS) was derived, as indicator of adherence to dietary recommendations [26] on fruits and vegetables intake, whole-meal foods, fish, refined sugars and fat intakes. The HDAS ranged from 0 to 50 and dichotomized as high (median ≥ 20) vs. low (median < 20) diet quality. These variables were considered due to the close relationship with metabolic health and screen-time in children [5]. For participants with available accelerometer data (W1: *N* = 4640, W2: *N* = 4344, W3 = 3238), daily moderate-to-vigorous physical activity (MVPA) and sedentary-time (SED) was measured using Actigraph accelerometers (Actigraph, LLC, Pensacola, FL, USA). The valid accelerometer wear-time (≥ 6 h/day) and total time spent in ≥ 30 min SED-bouts (derived allowing 2 min. of accumulated activities within 30 min. of sedentary time according to Evenson et al. cut-point [27]) and ≥ 10 min MVPA-bouts was calculated. These cut-offs were selected because: i) ≥ 10 min MVPA-bouts have been shown to confer benefits in children’s cardio-metabolic health [28]; ii) ≥ 30 min SED-bouts facilitates comparison with previous studies conducted in the same age range [29, 30]. The SED-time in bouts was categorized at median = 798 min/day as high vs. low SED-time. Regarding MVPA-time in bouts (median of any MVPA = 34 min/day), children were classified as: physically inactive (MVPA = 0 min/day), low MVPA (0 < MVPA ≤ 34 min/day) and high MVPA duration (> 34 min/day) in order to observe underlying differences between groups. As puberty influences physiological (e.g. hormonal changes), psychosocial and behavioral processes (e.g. sedentary patterns) [31], children aged ≥ 8 years (at W3) provided information on puberty status as: changes in voice (boys) and onset of menarche (girls) [32]. In a smaller sample (*N* = 2999), information on pubertal Tanner stage: pubic hair (boys) and breast development (girls) was obtained to complement the information on puberty [33]. Highest parental educational attainment was self-reported and classified according to the International Standard Classification of Education (ISCED) [34] as high, medium and low ISCED. Further details on covariates are given in the Supplementary file.

### Statistical analyses

Descriptive characteristics of the analysis population were generated (number and percentage) by sex and study wave at the most recent measurement point (W2 or W3). Missing values for HDAS, snacking frequency, pubertal status and ISCED were treated as an additional category (i.e. included in the analyses as missing category) to make better use of data provided on outcomes and exposure. Characteristics of participants excluded were compared to those included in the analysis population (eTable 1).

To investigate the role of DM use over time on MetS (and its components), a two-step trajectory analysis approach was used. This approach allows comparisons of individuals’ DM use over the age-span of the cohort, thus evaluating changes in DM duration (hours/day) with increasing age such that each child has its individual DM trajectory. This approach handles DM assessments at different time points and unbalanced data with different number of repeated measures per child as well as subjects measured at different ages [35–37].

#### First step: Linear DM trajectories over the age-span of the cohort

Trajectories of DM duration over age (2 to 16 years, centred at age 8) were estimated using linear mixed models including two levels (repeated measurements nested within individuals) to reduce data dimensionality and to derive exposure measures that are comparable between children. Models considered a random intercept and random linear slope over age per each child. To account for repeated measurements, the subject-specific DM intercepts and slopes were estimated from fixed and random effects. The random DM intercept (hours/day) and slope (hours/day/year) indicate the deviations for child *ί* from the average DM use across childhood (2–16 years) and from the average velocities (slopes) of DM increase over the age span (between 2–16 years), respectively. A detailed description of the mixed models is provided in the Supplementary material. Further, to investigate a fanning pattern and possible multicollinearity of random intercept and random slope, we calculated the covariance of both subject-specific parameters and further considered the tolerance and variance inflation factor (VIF) in regression models of step 2. Covariance was almost zero and did not indicate any fanning pattern as well as tolerance and VIF did not show any multicollinearity in regression models of step 2, particularly for random intercept and random slopes (results not shown). Age-dependent trajectories were additionally calculated by sex and country of residence (i.e. model was respectively stratified on sex and country, thus considering sex- and country-specific population intercept and slope), in order to take into account country- and sex-specific DM habits.

#### Second step: DM trajectories in association with MetS

The estimated individual DM intercepts and slopes were used as exposure variables in the longitudinal association with z-scores of MetS, WC, BP, HOMA-IR, HDL-c and triglycerides, at the most recent examination (W2 or W3, i.e. the highest age of each individual within the cohort). Generalized linear mixed regressions without a random effect were used to estimate regression coefficients (β) and 95% confidence intervals (95%CI), adjusting for confounders from the most recent examination: continuous age, sex, puberty status (ref. pre-pubertal), ISCED (ref. high), snack intake (high vs. low), HDAS (high vs. low); country as well as observation period (the difference between age at last follow-up and age at baseline), and baseline z-score of the respective outcome. When adiposity was not part of the outcome (i.e. BP, HOMA-IR, triglycerides, HDL-c), models were further adjusted for current WC z-score. Due to missing values for different components, sample size varied. These analyses were repeated in the sample with accelerometer data to observe if the association between DM trajectories and MetS attenuates in these children. At a later step, the role of physical activity was considered by further adjusting for MVPA- and SED-time in bouts (and accelerometer wear time).

The role of DM exposure over time on the risk of developing MetS (monitoring level) and its components was further investigated. The slopes of DM trajectories were dichotomized at the population mean (random slope = 0), to identify children with increasing DM above or below the average. Logistic regressions were used to estimate odds ratios (OR) and 95% CI adjusting for individual continuous intercept and confounders, except PA. Children being at monitoring level (≥ 90^th^ percentile) for MetS, abdominal obesity, BP, IR, and dyslipidaemia at baseline, were excluded from the respective analyses, in order to evaluate the long-term role of DM trajectories in the incident MetS and its components. The sample size varied due to missing values on single components.

### Additional analyses

The association of DM slope (categorized) with MetS was further investigated stratifying by sex, to observe sex-specific differences, and by country, to account for cross-country discrepancies. In a sensitivity analysis, we stratified the analysis group by parental ISCED to evaluate a potential interaction in the relationship between DM trajectories and MetS, as observed previously [38, 39]. Level of significance was set to α ≤ 0.05, without adjusting for multiple testing. Statistical analyses were conducted using SAS 9.4 (Statistical Analyses System, SAS Institute Inc., Cary, NC, USA).

## Results

**Fig. 1:**
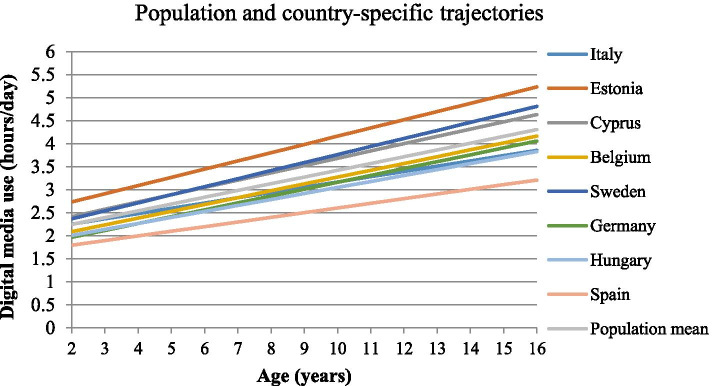
Country-specific digital media use trajectories in European children and adolescents

**Fig. 2:**
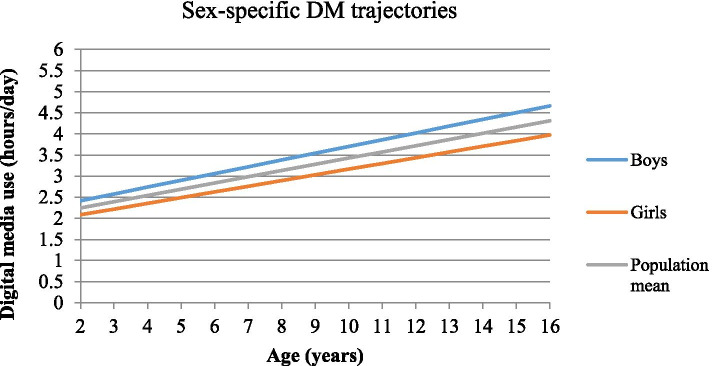
Sex-specific digital media use trajectories in European children and adolescents

A total of 10,359 children (50.3% girls), aged 2–16 years (mean = 7.9, SD = 2.7), with at least two observations were eligible (in total: 24,075 observations- described in eTable 2 by sex and examination wave). The excluded participants (eTable 1) were mostly boys, less than 12 years of age and pre-pubertal, with missing information on parental ISCED, diet quality and unhealthy snack intake frequency. A quarter (25%) of the excluded children and adolescents were from Cyprus. The characteristics of the analysis group at the last follow-up are described in Table 1. Overall, DM exposure increased as children grew older (Fig. 1), from 2.2 h/day at age 2 to 4.3 h/day at age 16 (mean intercept = 1.95 h/day, mean slope = 0.14 h/day/year). Boys developed a steeper DM increase compared to girls (Fig. 2). Estonian children showed the steepest increase (2.7 h/day at age 2 to 5.2 h/day at age 16), followed by Swedish and Cypriot children which were all above the average. Spanish children showed the lowest DM increase (1.8 h/day at age 2 to 3.2 h/day at age 16). Of all children, 28.7% suffered from abdominal obesity, 13.5% from dyslipidemia, 15.6% from IR, 17.4% showed elevated BP, and 5.5% were classified with MetS (monitoring level) (Table 1).

**Table 1:** Metabolic risk profiles and characteristics of analysis population at the most recent examination point

	Most recent examination point				All
	W2		W3		
	**Sex**				
	**Boys**	**Girls**	**Boys**	**Girls**	
	**N (%)**	**N (%)**	**N (%)**	**N (%)**	**N (%)**
**All**	2550 (24.6)	2543 (24.5)	2594 (25.0)	2672 (25.8)	10,359 (100.0)
**DM trajectory**					
Below mean	1274 (12.3)	1249 (12.1)	1312 (12.7)	1546 (14.9)	5381 (51.9)
Above mean	1276 (12.3)	1294 (12.5)	1282 (12.4)	1126 (10.9)	4978 (48.1)
**Age group**					
< 12 years	2550 (24.6)	2543 (24.5)	1410 (13.6)	1428 (13.8)	7931 (76.6)
≥ 12 years	0 (0)	0 (0)	1184 (11.4)	1244 (12.0)	2428 (23.4)
**ISCED**^**a**^					
Low	143 (1.4)	121 (1.2)	147 (1.4)	139 (1.3)	550 (5.3)
Medium	1088 (10.5)	1089 (10.5)	1104 (10.7)	1160 (11.2)	4441 (42.9)
High	1297 (12.5)	1309 (12.6)	1323 (12.8)	1353 (13.1)	5282 (51.0)
Missing	22 (0.2)	24 (0.2)	20 (0.2)	20 (0.2)	86 (0.8)
**HDAS**					
High	1336 (12.9)	1395 (13.5)	1085 (10.5)	1114 (10.8)	4930 (47.6)
Low	1097 (10.6)	1013 (9.8)	1426 (13.8)	1469 (14.2)	5005 (48.3)
Missing	117 (1.1)	135 (1.3)	83 (0.8)	89 (0.9)	424 (4.1)
**Snack intake**					
High	1012 (9.8)	967 (9.3)	1643 (15.9)	1610 (15.5)	5232 (50.5)
Low	1263 (12.2)	1261 (12.2)	723 (7.0)	858 (8.3)	4105 (39.6)
Missing	275 (2.7)	315 (3.0)	228 (2.2)	204 (2.0)	1022 (9.9)
**Puberty status**					
Pre-pubertal	1123 (10.8)	999 (9.6)	1387 (13.4)	1514 (14.6)	5023 (48.5)
Pubertal	0 (0.0)	0 (0.0)	1040 (10.0)	1035 (10.0)	2075 (20.0)
Missing	1427 (13.8)	1544 (14.9)	167 (1.6)	123 (1.2)	3261 (31.5)
**Country**					
Italy	326 (3.1)	297 (2.9)	530 (5.1)	514 (5.0)	1667 (16.1)
Estonia	269 (2.6)	301 (2.9)	403 (3.9)	444 (4.3)	1417 (13.7)
Cyprus	327 (3.2)	333 (3.2)	524 (5.1)	509 (4.9)	1693 (16.3)
Belgium	311 (3.0)	303 (2.9)	103 (1.0)	126 (1.2)	843 (8.1)
Sweden	366 (3.5)	361 (3.5)	295 (2.8)	307 (3.0)	1329 (12.8)
Germany	209 (2.0)	215 (2.1)	354 (3.4)	358 (3.5)	1136 (11.0)
Hungary	359 (3.5)	380 (3.7)	205 (2.0)	220 (2.1)	1164 (11.2)
Spain	383 (3.7)	353 (3.4)	180 (1.7)	194 (1.9)	1110 (10.7)
**Abdominal adiposity**					
No	1875 (18.1)	1831 (17.7)	1754 (16.9)	1867 (18.0)	7327 (70.7)
Yes	664 (6.4)	708 (6.8)	821 (7.9)	781 (7.5)	2974 (28.7)
Missing	11 (0.1)	4	19 (0.2)	24 (0.2)	58 (0.6)
**Elevated BP**					
No	1944 (18.8)	2037 (19.7)	2109 (20.4)	2209 (21.3)	8299 (80.1)
Yes	548 (5.3)	443 (4.3)	419 (4.0)	390 (3.8)	1800 (17.4)
Missing	58 (0.6)	63 (0.6)	66 (0.6)	73 (0.7)	260 (2.5)
**Dyslipidaemia**					
No	1425 (13.8)	1406 (13.6)	1542 (14.9)	1599 (15.4)	5972 (57.7)
Yes	385 (3.7)	408 (3.9)	315 (3.0)	292 (2.8)	1400 (13.5)
Missing	740 (7.1)	729 (7.0)	737 (7.1)	781 (7.5)	2987 (28.8)
**Insulin resistance**					
No	1599 (15.4)	1520 (14.7)	1480 (14.3)	1504 (14.5)	6103 (58.9)
Yes	442 (4.3)	496 (4.8)	332 (3.2)	342 (3.3)	1612 (15.6)
Missing	509 (4.9)	527 (5.1)	782 (7.5)	826 (8.0)	2644 (25.5)
**MetS**					
No	1607 (15.5)	1605 (15.5)	1627 (15.7)	1668 (16.1)	6507 (62.8)
Yes	159 (1.5)	165 (1.6)	127 (1.2)	117 (1.1)	568 (5.5)
Missing	784 (7.6)	773 (7.5)	840 (8.1)	887 (8.6)	3284 (31.7)

^a^ *W2* second wave of follow-up, *W3* third examination wave, *DM* digital media, *ISCED* parental educational status, *HDAS* healthy diet adherence score (diet quality), *BP* blood pressure, *MetS* metabolic syndrome

The regression results (Table 2) showed positive association between DM intercept (h/day) and slope (h/day/year) and WC z-score (intercept: β = 0.15, 95%CI = 0.11, 0.19; slope: β = 0.19; 95%CI = -0.04, 0.43). DM trajectories were positively associated with the later MetS z-score (intercept: β = 0.07, 95%CI = 0.02, 0.13; slope: β = 0.54, 95%CI = 0.20, 0.88), indicating that one hour increase in DM over time increased the MetS-score with 0.54. The repeated analysis in children with accelerometer data showed similar results. Further adjustment for MVPA and SED-time did not attenuate the associations, indicating positive associations between both DM intercept and slope and the later MetS z-score. However, larger confidence intervals were observed, due to the lower sample size. Positive associations were also observed between DM intercept and slopes and z-scores of BP, HOMA-IR and triglycerides, while inverse associations were observed with HDL-c z-score.

**Table 2:** Association of average DM across childhood (intercept) and increase of DM over time (slope) with metabolic syndrome score and its components in children and adolescents

**Metabolic outcomes**	**DM use**			**Analysis population**^**a**^		**Accelerometer –sample **^**b**^		
		**N**	**Crude β (95%CI)**	**N**	**Adjusted β (95%CI) **^**a**^	**N**	**Adjusted β (95% CI) **^**b**^	**Adjusted β (95% CI) **^**c**^
**z_WC **^**d**^	**Intercept**	10,301	**0.33 (0.28, 0.39)**	10,153	**0.15 (0.11, 0.19)**	4258	**0.19 (0.13, 0.26)**	**0.19 (0.12, 0.25)**
	**Slope**		-0.05 (-0.40, 0.29)		0.19 (-0.04, 0.43)		0.26 ( -0.10, 0.63)	0.26 (-0.10, 0.63)
**z_BP **^**e**^	**Intercept**	10,099	**0.04 (0.01, 0.08)**	9409	**0.04 (0.00, 0.07)**	4073	0.02 (-0.03, 0.08)	0.02 (-0.03, 0.07)
	**Slope**		-0.05 (-0.27, 0.16)		0.09 (-0.10, 0.30)		0.18 (-0.13, 0.49)	0.16 (-0.14, 0.47)
**z_TRG**	**Intercept**	7398	**0.11 (0.07, 0.15)**	6193	**0.08 (0.03, 0.12)**	2683	0.06 (-0.01, 0.13)	0.06 (-0.01, 0.13)
	**Slope**		0.17 (-0.08, 0.44)		0.02 (-0.24, 0.30)		0.01 (-0.40, 0.42)	0.00 (-0.41, 0.41)
**z_HDL-c**	**Intercept**	7766	**-0.10 (-0.14, -0.06)**	6506	**-0.08 (-0.12, -0.04)**	2857	**-0.07 (-0.14, -0.002)**	**-0.07 (-0.14, -0.00)**
	**Slope**		**-0.35 (-0.63, -0.06)**		**-0.28 (-0.54, -0.02)**		-0.32 (-0.72, 0.07)	-0.33 (-0.73, 0.06)
**z_HOMA**	**Intercept**	6293	**0.16 (0.11, 0.22)**	3435	**0.12 (0.05, 0.19)**	1688	**0.15 (0.05, 0.26)**	**0.15 (0.04, 0.25)**
	**Slope**		0.19 (-0.13, 0.51)		0.64 (0.21, 1.08)		0.58 (-0.01, 1.18)	0.59 (0.00, 1.19)
**z_MetS**	**Intercept**	5770	**0.16 (0.11, 0.21)**	2973	**0.07 (0.02, 0.13)**	1476	0.07 (-0.01, 0.15)	0.06 (-0.02, 0.14)
	**Slope**		0.14 (-0.15, 0.44)		**0.54 (0.20, 0.88)**		**0.51 ( 0.04, 0.97)**	**0.49 (0.02, 0.95)**

^a^ Models are adjusted for age (continuous), sex, pubertal status, HDAS, snack consumption, parental ISCED, observation period, (age at follow-up – age at baseline), country and baseline z-scores of the respective outcome. Bold significance is provided via confidence limits

^b^ Models are based on the accelerometer sample and are adjusted for same confounders as in the main analysis. N varied due to missing values for each outcome

^c^ Models based on sample with accelerometer data are further adjusted for MVPA, SED and valid accelerometer wear time

^d^ WC- waist circumference, BP-blood pressure, TRG- triglycerides, HDL-c– high density lipoprotein cholesterol, HOMA- homeostasis model assessment for insulin resistance, MetS- metabolic syndrome, DM- digital media

^**e**^ Models for the z-scores of BP, HDL-c, TRG and HOMA-IR are additionally adjusted for z-score of WC at the last measurement point. The number of participants varied for metabolic outcomes due to missing values

The logistic regression based on DM slope categories (Table 3) showed that children with increasing DM use above average had 30% higher risk of developing MetS (OR = 1.30, 95%CI = 1.04–1.62). This risk was higher in children with more educated parents (high ISCED: OR = 1.56, 95%CI = 1.07–2.26; medium: OR = 1.22, 95%CI = 0.90–1.66, low: OR = 0.92, 95%CI = 0.45–1.86) (eTable 3). Boys with increased DM above average had higher risk for elevated BP and IR, and 62% higher risk for MetS (OR = 1.62, 95%CI = 1.17–2.24). One hour increase in DM intercept was positively associated with MetS, abdominal obesity and IR in both sexes; stronger associations were observed for elevated BP and dyslipidemia in boys, compared to girls.

**Table 3:** Risk of metabolic syndrome and its components by DM slope and DM intercept in children and adolescents

**Metabolic outcomes **^**a**^	**DM use**		**Analysis Population**				**Boys**		**Girls**
		**N**	**Unadjusted OR (95% CI)**	**N**	**Adjusted OR (95% CI) **^**b**^	**N**	**Adjusted OR (95% CI) **^**c**^	**N**	**Adjusted OR (95% CI) **^**c**^
**Abdominal obesity**	**Slope **^**d**^	8114	1.00 (0.89–1.13)	7966	1.05 (0.92–1.19)	3966	0.97 (0.81–1.16)	4000	1.12 (0.94–1.34)
	**Intercept**		**1.49 (1.32–1.67)**		**1.53 (1.35–1.75)**		**1.30 (1.09–1.56)**		**1.85 (1.53–2.24)**
**High BP **^**e**^	**Slope**	8425	1.01 (0.89–1.14)	7693	1.04 (0.91–1.20)	3809	1.13 (0.94–1.36)	3884	0.96 (0.78–1.17)
	**Intercept**		**1.18 (1.04–1.33)**		1.08 (0.94–1.25**)**		1.15 (0.96–1.38)		1.01 (0.81–1.26)
**Dyslipidemia**	**Slope**	6248	1.07 (0.93–1.23)	5001	1.00 (0.85–1.18)	2469	1.04 (0.82–1.32)	2532	0.93 (0.73–1.17)
	**Intercept**		**1.30 (1.14–1.48)**		**1.28 (1.08–1.51)**		**1. 42 (1.13–1.78)**		1.08 (0.84–1.39)
**Insulin resistance**	**Slope**	6797	0.96 (0.85–1.09)	5435	1.00 (0.87–1.16)	2728	**1.22 (1.00–1.50)**	2707	0.83 (0.68–1.02)
	**Intercept**		**1.35 (1.20–1.52)**		**1.16 (1.00–1.35)**		1.15 (0.93–1.41)		1.12 (0.90–1.41)
**MetS**	**Slope**	6843	**1.21 (1.01–1.46)**	5288	**1.30 (1.04–1.62)**	2636	**1.62 (1.17–2.24)**	2652	1.08 (0.80–1.47)
	**Intercept**		**1.55 (1.30–1.84)**		**1.50 (1.21–1.85)**		**1.58 (1.20–2.11)**		1.35 (0.97–1.87)

^a^ The reference category for the metabolic outcomes is below the monitoring level

^b^ Models are adjusted for age (continuous), sex, pubertal status, country, parental ISCED, HDAS, snack frequency intake, observation period, and abdominal obesity (when BP, IR and dyslipidemia were modeled). Bold significance is provided via confidence limits

^c^ Models are adjusted for all covariates, besides sex (and physical activity variables)

^d^ Slope was used as a categorical variable (above vs. below population mean random slope)

^e^ BP-blood pressure, MetS- metabolic syndrome, DM- digital media

The country-stratified analyses are presented in Table 4. In Cyprus, children with increased DM use above average had two-fold higher risk of developing MetS (OR = 2.66, 95%CI = 1.38–5.14); while positive associations were observed for dyslipidemia (OR = 1.66, 95%CI = 1.05–2.63) and IR (OR = 1.45, 95%CI = 0.96–2.16). In Estonia and Sweden- also countries with above average DM trajectories- children had increased risk of developing abdominal obesity and MetS; Belgian children showed almost two-fold higher risk of developing elevated BP (OR = 1.87; 95%CI = 1.16–3.02) and MetS (OR = 2.08, 95%CI = 0.37–11.58). In Hungary, children with increased slope had higher risk for MetS, elevated BP and abdominal obesity. Remarkably, increasing DM intercept showed higher risk for MetS and its components across all countries, except Italy.

**Table 4:** Age-dependent digital media use trajectories by country of residence and risk of developing metabolic syndrome ^a^

Metabolic outcome ^b^	DM use	Italy	Estonia	Cyprus	Belgium	Sweden	Germany	Hungary	Spain
	**Mean intercept (h/day)**	2.0258	2.3790	2.1022	1.7932	2.0210	1.6706	1.7521	1.5931
	**Mean slope (h/day/year)**	0.1144	0.1785	0.1583	0.1483	0.1746	0.1493	0.1298	0.1012
		**Adjusted odds ratios (OR) and 95% confidence intervals (95% CI)**							
**Abdominal obesity**	**Slope **^**c**^	0.90 (0.68–1.20)	1.21 (0.86-1.70)	1.03 (0.75–1.41)	0.74 (0.46–1.20)	**1.69 (1.05–3.23)**	0.93 (0.63–1.36)	1.31 (0.90–1.92)	0.99 (0.67–1.46)
	**Intercept**	1.28 (0.96–1.69)	**1.62 (1.17–2.23)**	**1.53 (1.11–2.09)**	**2.07 (1.21–3.54)**	**1.84 (1.05–3.23)**	**1.78 (1.24–2.57)**	1.13 (0.75–1.73)	**2.11 (1.32–2.38)**
**Elevated BP **^**d**^	**Slope**	1.28 (0.94–1.74)	0.98 (0.68–1.41)	1.12 (0.73–1.70)	**1.87 (1.16–3.02)**	0.68 (0.40–1.18)	0.79 (0.47–1.32)	1.17 (0.84–1.64)	0.71 (0.49–1.03)
	**Intercept**	0.96 (0.72–1.28)	1.28 (0.90–1.81)	1.24 (0.83–1.85)	1.66 (0.99–2.80)	1.04 (0.52–2.07)	0.86 (0.50–1.47)	1.05 (0.73–1.50)	0.88 (0.55–1.41)
**Dyslipidemia**	**Slope**	0.96 (0.67–1.17)	1.00 (0.63–1.58)	**1.66 (1.05–2.63)**	0.61 (0.18–2.04)	1.19 (0.73–1.94)	0.86 (0.50–1.50)	0.86 (0.56–1.30)	0.77 (0.45–1.30)
	**Intercept**	1.10 (0.80–1.52)	1.46 (0.94–2.28)	1.09 (0.69–1.73)	2.37 (0.52–10.8)	1.72 (0.92–3.19)	1.37 (0.80–2.33)	1.30 (0.83–2.03)	1.37 (0.75–2.52)
**Insulin resistance**	**Slope**	0.86 (0.61–1.22)	1.36 (0.92–2.01)	1.45 (0.96–2.16)	0.73 (0.40–1.36)	0.94 (0.63–1.40)	1.05 (0.63–1.75)	0.81 (0.53–1.22)	0.98 (0.64–1.50)
	**Intercept**	0.84 (0.60–1.17)	1.42 (0.97–2.08)	1.20 (0.80–1.80)	1.51 (0.72–3.15)	**1.87 (1.12–3.12)**	1.58 (0.95–2.62)	1.12 (0.70–1.77)	0.73 (0.43–1.24)
**MetS**	**Slope**	0.99 (0.66–1.49)	1.69 (0.87–3.23)	**2.66 (1.38–5.14)**	2.08 ( 0.37–11.58)	1.29 (0.54–3.08)	0.82 (0.25–2.66)	1.34 (0.74–2.41)	0.91 (0.48–1.73)
	**Intercept**	0.95 (0.66–1.38)	1.78 (0.97–3.24)	1.66 (0.94–2.83)	1.14 (0.14–8.86)	**2.78 (1.04–7.41)**	**4.35 (1.68–11.29)**	**2.38 (1.33–4.25)**	1.57 (0.76–3.25)

^a^ Models are adjusted for age (continuous) sex, pubertal status, parental ISCED, HDAS, unhealthy snack intake, observation period and abdominal obesity (when not part of the outcome). The number of participants varied across countries due to missing values for different metabolic outcomes. Bold significance is provided via confidence limits

^b^ The reference category for the metabolic outcomes is below the monitoring level

^c^ Slope was used as a categorical variable (above vs. below population mean random slope)

^d^ *BP* blood pressure, *DM* digital media, *MetS* metabolic syndrome

## Discussion

### Key findings

In children of the IDEFICS/I.Family cohort, DM exposure increased with age, from 2.2 h/day at age 2 to 4.3 h/day at age 16. Estonian children showed the strongest DM increase while Spanish children showed the weakest. The average DM exposure across childhood (intercept) and increase of DM over time (slope- i.e. DM trajectory) were independently associated with the later z-scores of MetS and its components. Children with increased DM trajectories showed higher risk of developing MetS later in life.

These findings build upon previous cross-sectional studies where screen-time was positively associated with MetS [11, 40, 41]. Earlier investigations on IDEFICS children showed that DM use increased the risk for IR after two years [42], and having a media device in child’s bedroom increased the odds for abdominal obesity and MetS [43]. In our analysis, the associations of DM trajectories with z-scores of MetS, WC, BP, HOMA-IR and HDL^−1^ remained after adjustment for MVPA and SED-time, supporting previous findings [44]. One underlying explanation could be that sedentary screen-time in children is associated with lower metabolic rate (i.e. energy expenditure) compared to rest condition [45]. Further, children might engage with screen-based and MVPA-based activities simultaneously (e.g. exposure to age inappropriate advertisements such as those with violent content or for unhealthy foods while dancing to a music video on the internet) [46], thus undermining the positive effects of MVPA on metabolic health. These findings also shed light on a methodological aspect whereby digital media exposure is associated with metabolic syndrome independently of total sedentary time, thus they should not be interchangeably used, supporting previous concerns on examining different types of sedentary behaviors in relation with health outcomes [47].

### DM trajectories and risk of developing MetS—differences by sex

Children with DM trajectory above average showed higher risk of developing MetS, abdominal obesity and elevated BP. Both boys and girls with increasing average DM (intercept) showed an increased risk of developing MetS (58% in boys and 35% in girls), IR and abdominal obesity, indicating that a high, although stable DM use can deteriorate children’s metabolic outcomes in the long-term, independently of sex, supporting previous evidence [11, 44]. An increased risk for elevated BP and dyslipidemia as average DM increased was found only in boys, but not in girls. Boys also showed a steeper DM trajectory compared to girls. Previous evidence showed that boys are more likely to develop an increasing media use trajectory than girls [38]. A previous study reported that boys compared to girls, had higher screen-time, systolic BP and triglycerides, while lower HDL-c levels [10]. Furthermore, male sex has been described as a risk factor for childhood to early-midlife BP trajectories [48]. The different mechanisms of self-regulation and its role on health may provide further explanation. Digital media use (TV and mobile device) [49] is associated with poor self-regulation in children (e.g. inhibitory control, frustration tolerance), which in turn tends to be lower for boys than for girls [50]. Lower self-regulation in children increases the risk for elevated BP and cholesterol [51], as well as higher levels of stress [52]. A previous study based on our cohort [53] showed that lower psychological well-being was associated with cardio-metabolic disturbances. These data underline the importance that more efforts should be undertaken by physicians and parents to reduce DM use in boys, especially limiting (online) video-game use, which yet remains the most common screen-based activity among boys [54].

### DM trajectories and risk of developing MetS—differences by country

DM duration above the average (slope) increased the risk of developing metabolic disorders in countries with the steepest DM trajectory- Cyprus and Sweden. Nevertheless, increasing average DM consistently increased the risk for MetS in all countries. Clear differences were observed between northern (Estonia, Sweden) and southern countries (Spain) on DM trajectories, which could be due to the different cultures in handling DM exposure in children. In Northern countries, a media-rich bedroom culture is more common in comparison with southern countries, i.e. children and adolescents have their own bedrooms installed with a TV set, game console, and PC [55] which raises concerns about parent’s ability to control and regulate their children’s media use. Moreover, differences in parental digital literacy between countries may also relate to the parenting role in childhood DM exposure [54]. However, no clear patterns were observed on the risk of developing MetS, indicating that globalization of DM exposure influences children’s health independently of cultural/geographical differences.

### Clinical relevance and recommendations

Evidence suggests that prevention, early identification and control of cardio-metabolic risk factors throughout childhood, to adolescence and into adulthood will substantially reduce clinical risk for cardio-metabolic diseases beginning in young adult life [56]. Our study shows that prolonged childhood DM exposure is an independent risk factor for metabolic syndrome and its components at later stages of life and may thus contribute to the development of MetS over time. In light of the current COVID-19 pandemic, these findings are of utmost importance. The implemented policies (e.g. school closures, lockdown) have led to higher screen-time in children [57, 58]. Clinicians and health authorities should educate families in developing effective family media use plans [59] in order to reduce excessive screen-time and prevent future health emergencies. Clinicians, who are perceived as credible messengers for health information, should incorporate the history of child’s media use in their routine health maintenance visits as they do for nutrition or tobacco exposure, and provide personalized, age-specific advice to limit DM exposure, as also recommended by the American Academy of Pediatricians [60]. Among the strategies that parents may incorporate include: to take DM devices (e.g. TV and PC/game console) out of the child’s bedroom [47]; to supervise their children’s DM use and take advantage of the new tools (i.e. parental controlling apps) that monitor the content children are exposed to in their mobile devices; and model a healthy DM use themselves [61].

## Limitations and strengths

Our study has some methodological limitations. DM exposure was proxy-reported by parents of young children and self-reported by adolescents, thus we cannot exclude a social-desirability bias. Additionally, DM use patterns have changed since W1 (2007). TV has been replaced by use of smartphones and social media platforms, and we could not consider the influence of these newer media types on MetS. At W3, a lower sample was contacted for participation in Belgium and Spain compared to other countries, as they received no full funding [62]. At baseline, the percentage of children providing venous blood was low especially in Cyprus (7.7%). This explains the high number of excluded Cypriot children (25%) from the final analysis population. The reason behind is that most parents were unable to accompany their children to the examination center. Moreover, the modular approach facilitated the possibility to opt out of single examinations. This explains the high proportion of subjects with missing data on diet variables in the excluded sample. External validity may be limited, but a potential selection bias cannot be ruled out, as the main aim of the IDEFICS/I.Family cohort was to identify the role of lifestyle factors on shaping health-related behaviors in children and adolescents by asking the whole population to attend, and not subjects suffering from a specific health condition, as is the case in clinical studies [15, 16]. Further, children’s weight status but not their media exposure was associated with attrition rate at follow-up [63]. Accelerometer-data were collected only for a sub-sample of children; hence we cannot draw conclusions about MVPA- and SED-time for the entire population. However, the results were not affected by selection bias, as the low participation was due to budgetary limitations that restricted the number of devices provided. Since type of sedentary behaviors was not recorded by accelerometers (e.g. screen-based SED) we could not objectively assess screen-time. Internet exposure was measured only at T3 and we did not distinguish between its access via a smartphone/tablet or computer. Current literature suggests that smartphones were the most popular devices children used to go online [64]. Future studies should investigate the ubiquitous exposure to internet via smartphones on children’s metabolic health. Further, due to the low number of repeated measures, we could not consider a change in DM slope around puberty, e.g. modelling an exponential or quadratic slope. Additionally, AVM latent profile / transition analysis was not considered [65], due to the high age range and the unbalanced data (two or three observations per participant) that could be handled with the linear mixed models.

To our best knowledge, this is the first study evaluating the longitudinal association of DM exposure with MetS in children and adolescents. The availability of fasting blood samples represents an advantage in evaluating objectively-measured metabolic risks. In comparison to most other studies, besides TV, we included computer and internet exposure, thus capturing a larger picture of DM patterns. The availability of objectively-measured MVPA reduced the level of misreporting due to socially-desirable answers on physical activity [66]. The information on various covariates (e.g. consumption frequency of snacks, parental ISCED), enabled us to control for confounders. The large sample size of 10,359 children from 8 European countries providing harmonized data, allowed us to evaluate country-differences on DM trajectories and its association with MetS.

## Conclusions

Increased digital media exposure over time is associated with higher risk for metabolic syndrome in children and adolescents, with boys being at higher risk. These findings are of relevance for clinicians and families and ask for action by health authorities. Future health policies should focus on the reduction of screen-time throughout childhood and starting at an early age to prevent cardio-metabolic diseases.

## Supplementary Information

**Additional file 1: eTable 1**. Characteristics of participants excluded from the analysis compared to those included in the analysis population. **eTable 2**. Metabolic risk profiles and characteristics of analysis population at each study wave^a^. **eTable 3**. Association of digital media use trajectories with metabolic syndrome and its components in children, stratified by parental educational status^a^

## Data Availability

Due to the sensitive nature of data collected, ethical restrictions prohibit the authors from making the minimal data set publicly available. Each cohort centre received approval of the corresponding local Ethical Commission and participants did not provide consent for data sharing. Data are available on request and all requests need approval by the study’s Steering Committee. Interested researchers can contact the study co-ordinator (ahrens@leibniz-bips.de) to request data access. All requests for accessing data of the IDEFICS/I.Family cohort are discussed on a case-by-case basis by the Steering Committee.

## Abbreviations

DM: Digital media use
BP: Blood pressure
HDAS: Healthy diet adherence score
HDL-c: High density lipoprotein cholesterol
HOMA: Homeostasis model assessment for insulin resistance
IDEFICS: Identification and prevention of Dietary- and lifestyle-induced health EFfects In Children and infantS
I.Family: Determinants of eating behavior in European children, adolescents and their parents
ISCED: International standard classification of education
IR: Insulin resistance
MetS: Metabolic syndrome
TRG: Triglycerides
WC: Waist circumference
