# Supplementary material for: Media use trajectories and risk of metabolic syndrome in European children and adolescents: the IDEFICS/I.Family cohort
Source: Int J Behav Nutr Phys Act. 2021 Oct 18;18:134. doi: 10.1186/s12966-021-01186-9 (PMC8521295; doi:10.1186/s12966-021-01186-9)
Supplement: Supplementary file 1 — Additional file 1: eTable 1. Characteristics of participants excluded from the analysis compared to those included in the analysis population. eTable 2. Metabolic risk profiles and characteristics of analysis population at each study wavea. eTable 3. Association of digital media use trajectories with metabolic syndrome and its components in children, stratified by parental educational statusa [file 12966_2021_1186_MOESM1_ESM.docx]

**Media use trajectories and risk of metabolic syndrome in European children and adolescents: the IDEFICS and I.Family cohort**

Elida Sina MSc.^1^, Christoph Buck PhD^1^, Toomas Veidebaum PhD^2^, Alfonso Siani MD^3^, Lucia Reisch Dr.oec^4^, Hermann Pohlabeln PhD^1^, Valeria Pala PhD^5^, Luis Moreno PhD^6^, Dénes Molnar PhD^7^, Lauren Lissner PhD^8^, Yiannis Kourides PhD^9^, Stefaan De Henauw PhD^10^, Gabriele Eiben PhD^11^, Wolfgang Ahrens PhD^1,12^, Antje Hebestreit PhD^1^ on behalf of the IDEFICS and I.Family consortia

**Dietary patterns**

To assess the diet quality, a healthy diet adherence score (HDAS) was developed. Using a food frequency questionnaire (FFQ), participants indicated the frequency of consumption of 59 different foods items, beverages and mixed dishes in a typical week during the preceding 4 weeks. Answer options varied from ‘never/less than once a week’, ‘1–3 times/week’, ‘4–6 times/week’, ‘1 time/day’, ‘2 times/day’, ‘3 times/day’ to ‘4 or more times/day’. The description of food items was standardized across countries; examples of country-specific foods were included for a certain food item, to account for cultural discrepancies of food intake. The score was calculated for children with ≥50% of non-missing food items. The HDAS was developed as a composite score to reflect the adherence to the healthy dietary guidelines common across all the participating countries including high consumption of fruits and vegetables (at least 400-500 grams/day), limited intake of refined sugars and fat (especially saturated fats), consumption of whole meals, and of fish for 2-3 times per week (1), as established by Waijers et al. (2007) (2). The score ranged from 0 to 50 and was dichotomized based on median value as “high” vs. “low” adherence to assess the broad concept of healthy diet adherence and to better interpret the data.

**Accelerometer derived data**

For children with available accelerometer data, total moderate-to-vigorous physical activity (MVPA) and sedentary time (SED) duration per day was measured using Actigraph accelerometers (Actigraph, LLC, Pensacola, FL, USA)- in IDEFICS (W1/W2) either ActiTrainer or GT1M devices were used, as both models have identical sensor units; while in I.Family (W3) either GT3X+ or GT1M devices. In W0 and W1, children were asked to wear the accelerometer for at least three days (two weekdays and one weekend day) (3) while in W3 for seven consecutive days (4). Children wore the accelerometers on the right hip (attached with an elastic belt) during waking hours, except when taking a shower or engaged in water-based activities (e.g. swimming) (5). Accelerometer data were included when PA was recoded for at least two valid weekdays and one valid weekend day and for a minimum of 6 hours/day (6) valid wear-time after exclusion of non-wear time according to Choi et al. (2011) (7).

**eTable 1. Characteristics of participants excluded from the analysis compared to those**

**included in the analysis population**

| **Characteristics** | **Analysis population** | | **Excluded sample** | |
| --- | --- | --- | --- | --- |
|  | N | % | N | % |
| **All** | 10359 | 100.0 | 5845 | 100.0 |
| **Sex** |  |  |  |  |
| Boys | 5144 | 49.6 | 3109 | 53.2 |
| Girls | 5215 | 50.3 | 2736 | 46.8 |
| **Age group** |  |  |  |  |
| Children | 7931 | 76.6 | 4949 | 84.7 |
| Adolescents | 2428 | 23.4 | 896 | 15.3 |
| **Weight Status** |  |  |  |  |
| Normal weight | 7755 | 74.9 | 4414 | 75.5 |
| Overweight | 1865 | 18.0 | 856 | 14.6 |
| Obese | 739 | 7.1 | 542 | 9.3 |
| Missing |  |  | 33 | 0.6 |
| **Parental education status** |  |  |  |  |
| Low | 550 | 5.3 | 347 | 5.9 |
| Medium | 4441 | 42.9 | 1995 | 34.1 |
| High | 5282 | 51.0 | 2055 | 35.2 |
| Missing | 86 | 0.8 | 1448 | 24.8 |
| **Puberty status** |  |  |  |  |
| Pre-pubertal | 5023 | 48.5 | 3604 | 61.7 |
| Pubertal | 2075 | 20.0 | 639 | 10.9 |
| Missing | 3261 | 31.5 | 1602 | 27.4 |
| **Unhealthy snack intake** |  |  |  |  |
| Low | 4105 | 39.6 | 1202 | 20.6 |
| High | 5232 | 50.5 | 2078 | 35.6 |
| Missing | 1022 | 9.9 | 2565 | 43.9 |
| **Diet quality** |  |  |  |  |
| High | 4930 | 47.6 | 1729 | 29.6 |
| Low | 5005 | 48.3 | 1958 | 33.5 |
| Missing | 424 | 4.1 | 2158 | 36.9 |
| **Country** |  |  |  |  |
| Italy | 1667 | 16.1 | 365 | 6.2 |
| Estonia | 1417 | 13.7 | 462 | 7.9 |
| Cyprus | 1693 | 16.3 | 1460 | 25.0 |
| Belgium | 843 | 8.1 | 993 | 17.0 |
| Sweden | 1329 | 12.8 | 461 | 7.9 |
| Germany | 1136 | 11.0 | 773 | 13.2 |
| Hungary | 1164 | 11.2 | 1011 | 17.3 |
| Spain | 1110 | 10.7 | 320 | 5.5 |

**Detailed explanation of the mixed model to derive the DM trajectories**

A two-step approach was used to investigate the longitudinal effect of changes in media use (DM) over age and metabolic markers at latest time point. This approach handles DM assessments at different time points and unbalanced data with different number of repeated measures per child as well as subjects measured at different ages (6, 8, 9). Moreover, these models allow for change in scale and variance of the exposure measurements over time.

- For the first step, estimated trajectories of DM duration over age from 2 to 16 years using linear mixed models regressing continuous age in years to continuous DM (in hours per day) with further specification:
  - Age was centred at age 8 to calculate average DM across age;
  - A two-level hierarchical cluster structure was considering by means of a random intercept for repeated measurement occasions nested within individuals on the residual side of the covariance to reduce data dimensionality and to derive exposure measures that are comparable between children;
  - Further, random intercept and linear slope over age were modelled to estimate parameters of trajectories for each child;
  - Trajectories were calculated for each country stratum.
- To prepare for the second step, the subject-specific DM intercepts and slopes were calculated from
  - population average (fixed) intercept and random intercept per subject;
  - and average (fixed) age-effect and random slope.
- In the second step, the calculated random coefficients (individual DM intercepts and slopes) were used as exposure variables in generalized linear mixed regression models to investigate the longitudinal association of AVM trajectories with z-scores of MetS, WC, BP, HOMA-IR, HDL-c and triglycerides, at the most recent examination point.

**eTable 2. Metabolic risk profiles and characteristics of analysis population at each study wave^a^**

|  | **Survey** | | | | | | | | | | | | | | **All** | |
| --- | --- | --- | --- | --- | --- | --- | --- | --- | --- | --- | --- | --- | --- | --- | --- | --- |
|  | **W1** | | | | | **W2** | | | | **W3** | | | | |  |  |
|  | **Sex** | | | | | | | | | | | | | |  |  |
|  | **Boys** | | **Girls** | | **Boys** | | | **Girls** | | | **Boys** | | **Girls** | |  |  |
|  | **n** | **%** | **n** | **%** | **n** | | **%** | **n** | **%** | | **n** | **%** | **n** | **%** | **n** | **%** |
| **All** | 4736 | 19.7 | 4765 | 19.8 | 4613 | | 19.2 | 4695 | 19.5 | | 2594 | 10.8 | 2672 | 11.1 | 24075 | 100.0 |
| **Age group** | 4736 | 19.7 | 4765 | 19.8 | 4613 | | 19.2 | 4695 | 19.5 | | 1410 | 5.9 | 1428 | 5.9 | 21647 | 89.9 |
| Children |  |  |  |  |  |  |  |  |  |  |  |  |  |  |  |  |
| Adolescents | . | . | . | . | . | | . | . | . | | 1184 | 4.9 | 1244 | 5.2 | 2428 | 10.1 |
| **ISCED** |  |  |  |  |  | |  |  |  | |  |  |  |  |  |  |
| Low | 271 | 1.1 | 275 | 1.1 | 249 | | 1.0 | 223 | 0.9 | | 147 | 0.6 | 139 | 0.6 | 1304 | 5.4 |
| Medium | 2036 | 8.5 | 2045 | 8.5 | 1914 | | 8.0 | 1972 | 8.2 | | 1104 | 4.6 | 1160 | 4.8 | 10231 | 42.5 |
| High | 2400 | 10.0 | 2415 | 10.0 | 2404 | | 10.0 | 2456 | 10.2 | | 1323 | 5.5 | 1353 | 5.6 | 12351 | 51.3 |
| Missing | 29 | 0.1 | 30 | 0.1 | 46 | | 0.2 | 44 | 0.2 | | 20 | 0.1 | 20 | 0.1 | 189 | 0.8 |
| **HDAS** |  |  |  |  |  | |  |  |  | |  |  |  |  |  |  |
| High | 2351 | 9.8 | 2419 | 10.0 | 2438 | | 10.1 | 2609 | 10.8 | | 1085 | 4.5 | 1114 | 4.6 | 12016 | 49.9 |
| Low | 2191 | 9.1 | 2124 | 8.8 | 1945 | | 8.1 | 1860 | 7.7 | | 1426 | 5.9 | 1469 | 6.1 | 11015 | 45.8 |
| Missing | 194 | 0.8 | 222 | 0.9 | 230 | | 1.0 | 226 | 0.9 | | 83 | 0.3 | 89 | 0.4 | 1044 | 4.3 |
| **Snack intake** |  |  |  |  |  | |  |  |  | |  |  |  |  |  |  |
| High | 2033 | 8.4 | 2057 | 8.5 | 1821 | | 7.6 | 1822 | 7.6 | | 1643 | 6.8 | 1610 | 6.7 | 10986 | 45.6 |
| Low | 2134 | 8.9 | 2133 | 8.9 | 2256 | | 9.4 | 2321 | 9.6 | | 723 | 3.0 | 858 | 3.6 | 10425 | 43.3 |
| Missing | 569 | 2.4 | 575 | 2.4 | 536 | | 2.2 | 552 | 2.3 | | 228 | 0.9 | 204 | 0.8 | 2664 | 11.1 |
| **Puberty status** |  |  |  |  |  | |  |  |  | |  |  |  |  |  |  |
| Pre-pubertal | 4071 | 16.9 | 4025 | 16.7 | 2228 | | 9.3 | 2085 | 8.7 | | 1387 | 5.8 | 1514 | 6.3 | 15310 | 63.6 |
| Pubertal | 0 | 0 | 0 | 0 | 0 | | 0 | 0 | 0 | | 1040 | 4.3 | 1035 | 4.3 | 2075 | 8.6 |
| Missing | 665 | 2.8 | 740 | 3.1 | 2385 | | 9.9 | 2610 | 10.8 | | 167 | 0.7 | 123 | 0.5 | 6690 | 27.8 |
| **Country** |  |  |  |  |  | |  |  |  | |  |  |  |  |  |  |
| Italy | 795 | 3.3 | 755 | 3.1 | 752 | | 3.1 | 709 | 2.9 | | 530 | 2.2 | 514 | 2.1 | 4055 | 16.8 |
| Estonia | 591 | 2.5 | 643 | 2.7 | 622 | | 2.6 | 688 | 2.9 | | 403 | 1.7 | 444 | 1.8 | 3391 | 14.1 |
| Cyprus | 721 | 3.0 | 714 | 3.0 | 742 | | 3.1 | 745 | 3.1 | | 524 | 2.2 | 509 | 2.1 | 3955 | 16.4 |
| Belgium | 379 | 1.6 | 398 | 1.7 | 402 | | 1.7 | 411 | 1.7 | | 103 | 0.4 | 126 | 0.5 | 1819 | 7.6 |
| Sweden | 653 | 2.7 | 651 | 2.7 | 620 | | 2.6 | 637 | 2.6 | | 295 | 1.2 | 307 | 1.3 | 3163 | 13.1 |
| Germany | 522 | 2.2 | 541 | 2.2 | 439 | | 1.8 | 447 | 1.9 | | 354 | 1.5 | 358 | 1.5 | 2661 | 11.1 |
| Hungary | 525 | 2.2 | 527 | 2.2 | 486 | | 2.0 | 530 | 2.2 | | 205 | 0.9 | 220 | 0.9 | 2493 | 10.4 |
| Spain | 550 | 2.3 | 536 | 2.2 | 550 | | 2.3 | 528 | 2.2 | | 180 | 0.7 | 194 | 0.8 | 2538 | 10.5 |
| **Abdominal Obesity** |  |  |  |  |  | |  |  |  | |  |  |  |  |  |  |
| No | 3693 | 15.3 | 3679 | 15.3 | 3409 | | 14.2 | 3410 | 14.2 | | 1754 | 7.3 | 1867 | 7.8 | 17812 | 74.0 |
| Yes | 973 | 4.0 | 1012 | 4.2 | 1186 | | 4.9 | 1274 | 5.3 | | 821 | 3.4 | 781 | 3.2 | 6047 | 25.1 |
| Missing | 70 | 0.3 | 74 | 0.3 | 18 | | 0.1 | 11 | 0.0 | | 19 | 0.1 | 24 | 0.1 | 216 | 0.9 |
| **High BP** |  |  |  |  |  | |  |  |  | |  |  |  |  |  |  |
| No | 3605 | 15.0 | 3668 | 15.2 | 3605 | | 15.0 | 3783 | 15.7 | | 2109 | 8.8 | 2209 | 9.2 | 18979 | 78.8 |
| Yes | 785 | 3.3 | 762 | 3.2 | 887 | | 3.7 | 781 | 3.2 | | 419 | 1.7 | 390 | 1.6 | 4024 | 16.7 |
| Missing | 346 | 1.4 | 335 | 1.4 | 121 | | 0.5 | 131 | 0.5 | | 66 | 0.3 | 73 | 0.3 | 1072 | 4.5 |
| **Dislipidemia** |  |  |  |  |  | |  |  |  | |  |  |  |  |  |  |
| No | 3086 | 12.8 | 3144 | 13.1 | 2718 | | 11.3 | 2767 | 11.5 | | 1542 | 6.4 | 1599 | 6.6 | 14856 | 61.7 |
| Yes | 698 | 2.9 | 656 | 2.7 | 711 | | 3.0 | 723 | 3.0 | | 315 | 1.3 | 292 | 1.2 | 3395 | 14.1 |
| Missing | 952 | 4.0 | 965 | 4.0 | 1184 | | 4.9 | 1205 | 5.0 | | 737 | 3.1 | 781 | 3.2 | 5824 | 24.2 |
| **IR** |  |  |  |  |  | |  |  |  | |  |  |  |  |  |  |
| No | 3171 | 13.2 | 3210 | 13.3 | 2958 | | 12.3 | 2901 | 12.0 | | 1480 | 6.1 | 1504 | 6.2 | 15224 | 63.2 |
| Yes | 536 | 2.2 | 533 | 2.2 | 786 | | 3.3 | 868 | 3.6 | | 332 | 1.4 | 342 | 1.4 | 3397 | 14.1 |
| Missing | 1029 | 4.3 | 1022 | 4.2 | 869 | | 3.6 | 926 | 3.8 | | 782 | 3.2 | 826 | 3.4 | 5454 | 22.7 |
| **MetS** |  |  |  |  |  | |  |  |  | |  |  |  |  |  |  |
| No | 3337 | 13.9 | 3370 | 14.0 | 3072 | | 12.8 | 3118 | 13.0 | | 1627 | 6.8 | 1668 | 6.9 | 16192 | 67.3 |
| Yes | 138 | 0.6 | 142 | 0.6 | 272 | | 1.1 | 280 | 1.2 | | 127 | 0.5 | 117 | 0.5 | 1076 | 4.5 |
| Missing | 1261 | 5.2 | 1253 | 5.2 | 1269 | | 5.3 | 1297 | 5.4 | | 840 | 3.5 | 887 | 3.7 | 6807 | 28.3 |

^a^HDAS - healthy diet adherence score (diet quality); ISCED- parental educational status; BP- blood pressure, IR- insulin resistance, MetS- metabolic syndrome

In a sensitivity analyses, we evaluated the association of DM slope categories above population average with metabolic syndrome (MetS) and its single components, considering a potential interaction with parental educational status (max ISCED) **(eTable 3).** After stratification by ISCED, the association between increased DM slope and MetS was stronger in children with more educated parents (high ISCED: OR=1.56, 95%CI=1.07-2.26; medium ISCED: OR=1.22, 95%CI=0.90-1.66, low ISCED: OR=0.92, 95%CI=0.45-1.86). Children with increased DM use above country average had higher odds for dyslipidemia if their parents had a high ISCED (OR=1.15, 95%CI=0.90-1.47). Interestingly, increasing average DM use across childhood (individual mean intercept) was positively and significantly related to MetS and its single components, independently of parental educational background.

**eTable 3. Association of digital media use trajectories with metabolic syndrome and its components in children, stratified by parental educational status^a^**

|  | **DM use** | **Odds ratios (OR) and 95% Confidence intervals (95% CI)** | | |
| --- | --- | --- | --- | --- |
|  |  | **Parental educational status** | | |
| **Outcome** |  | **High** | **Medium** | **Low** |
| **Abdominal obesity**  **High blood pressure ^b^**  **Dyslipidaemia ^b^**  **Insulin Resistance ^b^**  **Metabolic syndrome** | Slope  Intercept  Slope  Intercept  Slope  Intercept  Slope  Intercept | 1.12 (0.93-1.35)  **1.76 (1.43-2.16)**  0.89 (0.73-1.09)  **1.27 (1.02-1.59)**  1.15 (0.90-1.47)  **1.41 (1.07-1.87)**  1.08 (0.87-1.33)  1.15 (0.90-1.47) | 1.01 (0.84-1.21)  **1.41 (1.18-1.70)**  **1.24 (1.02-1.51)**  0.97 (0.80-1.20)  0.91 (0.71-1.15)  1.14 (0.90-1.44)  0.94 (0.76-1.16)  1.22 (0.98-1.50) | 0.73 (0.43-1.26)  1.38 (0.85-2.25)  0.88 (0.50-1.57)  0.91(0.55-1.51)  0.72 (0.38-1.36)  1.60 (0.92-2.79)  0.97 (0.54-1.72)  1.13 (0.66-1.95) |
|  | Slope  Intercept | **1.56 (1.07-2.26)**  **1.83 (1.23-2.72)** | 1.22 (0.90-1.66)  **1.46 (1.10-1.94)** | 0.92 (0.45-1.86)  1.24 (0.69-2.23) |

^a^ Models are adjusted for sex, age (continuous), pubertal status, HDAS, snack consumption, country, observation period. **^b^** Models are additionally adjusted for current waist circumference.

**Additional material references:**

1. Arvidsson L, Eiben G, Hunsberger M, De Bourdeaudhuij I, Molnar D, Jilani H, et al. Bidirectional associations between psychosocial well-being and adherence to healthy dietary guidelines in European children: prospective findings from the IDEFICS study. BMC Public Health. 2017;17(1):926.

2. Waijers PMCM, Feskens EJM, Ocké MC. A critical review of predefined diet quality scores. British Journal of Nutrition. 2007;97(2):219-31.

3. Konstabel K, Veidebaum T, Verbestel V, Moreno LA, Bammann K, Tornaritis M, et al. Objectively measured physical activity in European children: the IDEFICS study. Int J Obes. 2014;38(2):S135-S43.

4. Sprengeler O, Buck C, Hebestreit A, Wirsik N, Ahrens W. Sports Contribute to Total Moderate to Vigorous Physical Activity in School Children. Medicine and science in sports and exercise. 2019;51(8):1653-61.

5. Santaliestra-Pasías AM, Dios JEL, Sprengeler O, Hebestreit A, De Henauw S, Eiben G, et al. Food and beverage intakes according to physical activity levels in European children: the IDEFICS (Identification and prevention of Dietary and lifestyle induced health EFfects In Children and infantS) study. Public Health Nutrition. 2018;21(9):1717-25.

6. Buck C, Eiben G, Lauria F, Konstabel K, Page A, Ahrens W, et al. Urban Moveability and physical activity in children: longitudinal results from the IDEFICS and I.Family cohort. Int J Behav Nutr Phys Act. 2019;16(1):128.

7. Choi L, Liu Z, Matthews CE, Buchowski MS. Validation of accelerometer wear and nonwear time classification algorithm. Medicine and science in sports and exercise. 2011;43(2):357-64.

8. Börnhorst C, Siani A, Russo P, Kourides Y, Sion I, Molnár D, et al. Early Life Factors and Inter-Country Heterogeneity in BMI Growth Trajectories of European Children: The IDEFICS Study. PLOS ONE. 2016;11(2):e0149268.

9. Sayers A, Heron J, Smith A, Macdonald-Wallis C, Gilthorpe MS, Steele F, et al. Joint modelling compared with two stage methods for analysing longitudinal data and prospective outcomes: A simulation study of childhood growth and BP. Stat Methods Med Res. 2017;26(1):437-52.
